# 

**Published:** 1848-10-01

**Authors:** 


### INDEX TO VOL I.

ABERDEEN, report of Lunatic Asylum of, for 1847, 387.
———— remarks in, on the origin of insanity, 387, 388.
Acetate of morphia, in suicidal cases, 4, 5.
———— how administered in mania, 6.
Administration of medicine to the insane, remarks on, 395.
Administration of ether in treatment of insanity, 168.
Agency of the mind, 366—368.
Ages, table of periods of, when insanity commences, 89.
Agony experienced in reviving from drowning, 119.
Aix La Chapelle, dance of St John at, 412.
Allnutt, Boy, Baron Rolfe, charge of to grand jury, in case of, 193.
Ameliorated condition of modern lunatic asylums described, 31.
Amiens, cataleptic epidemy at, 655.
Anabaptists, early fanatical delusions of, 240—242.
Analysis, general, of dissections made at Bethlem Hospital, 63.
Andral, history of monomania of St. Soward, 421.
Anger, effects of described, 253.
Animal magnetism, as a means of relief in mania, 503—506.
Anodynes in puerperal mania, 286—288.
Anne, St., farm of, worked by the insane, 110.
Anecdote of Black, 118.
———— a Capuchin, 369.
———— Cowper, 237.
———— Cullen in his last moments, 118.
———— Archdeacon Nares, 15.
———— extraordinary of a lady and beggar, 203, 204.
Apparitions, on their psychology and physiology, 512.
Appetites, indulgence in, leads to extinction of reason, 492.
Appert, remarks of, on solitary system, 159.
Approach of mania and of consumption, contrasted, 88.
Aquinas, St. Thomas, on Spirit, 228.
Arrangement of mental affections, by the Baron Feuchtersleben, 261, 262.
Arson, mental alienation, 476.
Asylums, lunatic, general view of, on the continent, 94, 95.
———— royal, of Charenton, description of, 110, 111.
———— imperfect state of, in the Swiss Cantons, 96.
———— German, for the insane, reports and statutes of, 310, 313.
———— in Ireland, state of, 151.
———— New Middlesex Pauper Lunatic Asylum, 191.
———— in Norway, reports and statistics of, 313, 314.
———— Parisian, notes on, 100.
———— principal of Europe, sketch of, 252.
———— private, of MM. Falret and Voisin, 110.
———— private, former, atrocities perpetrated at, 30,
———— proposed for idiots, 190.
———— in Switzerland, 92.
Asylum in Vienna, Ball at, 168.
———— York, ancient horrors of, 29.
Attacks of insanity, period of year, at which they are most liable to occur, tabular view of, 89.
———— after, liability to in cases of puerperal mania, 151.
Attendants on lunatics, observations on, 82.
———— difficulties of their position, 82.

Attendants on lunatics, proportion of to the insane, 83.
——— Dr. Conolly on their qualifications, 84.
——— on their duties, 85, 86.
Augustus, Emperor, cured of melancholia by extract of lettuce, 5.

Baden, Grand Duchy of, statistics of suicide in, 315, 316.
Baillarger on hereditary insanity, 270.
Ball in the lunatic asylum at Vienna, 168.
Barlow, Dr., on instinctive insanity, 47.
Baron Rolfe, charge to the jury in case of boy Allnutt, 193.
Basting on solitary system for criminals, 159.
Baths and continuous affusion of water in acute mania, 456.
——— tabular view of, results, 457.
——— conclusion from, 457, 458.
Bedford lunatic asylum, report of, for 1847, 396.
Belfast, excess of mortality on side of females, 65.
Belhomme, M., on political and epidemic insanity, 406.
——— his conclusions, 425.
Belladonna atropa, effects of, 303—305.
——— singular action of, on a tailor, 304.
Berlin, university of, prize, question of, 169.
Bethlem, royal hospital for insane, report of, 400, 401.
——— tables, interesting, relating to, 403.
——— statistics of, by Dr. Webster, 58, 59.
——— number of cures, increase in, 59.
——— deaths in, gradually decline, 59.
——— statistical tables of, show mania more prevalent among women than men, 60.
——— statistical tables from, illustrating influence of seasons on insanity, 62.
——— seasons at which cures most prevalent, 62.
——— periods of greatest mortality in, 62.
——— dissections made at, 63.
——— average of weekly cases of restraint in, for the period of seven years, 109.
——— tabular record of duration of disease in puerperal cases, 149.
Bethlem hospital, as it was in 1816, 29.
——— mode of treatment followed out in, 29.
Bicêtre, idiots of, Dr. Sigmond on, 124.
Black, Dr., composure of, in his dying moments, 118.
Bleeding in puerperal mania, 285.
Blister, in cases of refusal to take food, successfully applied to the stomach, 11.
Blood, condition of, as a predisposing cause to insanity, 347.
Board, scale of, at royal asylum of Charenton, 111.
Body, use of, in relation to the mind, by Dr. Moore, 489.
Boismont, de Brierre, on the use of baths in mania, 456.
Brain, the human, by S. Solly, Esq., F.R.S., analysis of the work, 17, 18, 19.
——— human development of, 19.
——— action of stramonium on, 301, 302.
——— cannabis indica on, 297, 298, 299, 300, 301.
——— belladonna atropa on, 303, 304, 305.
——— hyoscyamus on, 305.
——— camphor on, 305, 306.
——— digitalis on, 306.
——— tobacco on, 306.
——— induration of, 359.
——— softening of, 359.
——— physical injuries to, induce indiscriminately all modes of mental disease, 155.
——— malformation of, by Dr. Wigan, and inferences, 306.
——— case of complete absorption of one hemisphere, by Dr. Conolly, 223.
——— disease of, symptoms of analogy between and action of certain drugs, 295, 296, 297, 298.
Brigham, Dr., on the pulse of the insane, 90.
——— on the physical character of the heads of the insane, 90.
Browne, Dr., on impairment of mind, 390, 391.
——— on suicide, 391, 392.
Broussais, on organic changes affecting the intellect, 421.
Brutalities, formerly enacted in lunatic asylums, harrowing account of, 30.
Brutus, feigned insanity of, doubtful, 278.
Burnett, Dr., on the cause of insanity, 541.
——— analysis of his book, 543, et seq.

Camp-meetings, scenes attending, 421.
Camphor, action of, on the brain, 305, 306.
Cannabis indica, effects of, as an hypnotic, 287, and 297—301.
Cantons, Swiss, state of lunatic asylums in the, 96, 99.
Capuchin friar, anecdote of, 369.
Carotids, on compression of, in headache, hysteria, epilepsy, &c., 626.
Cases of insanity, illustrative of, effects of acetate of morphia, 6, 7.
—— of compression of carotids, 629—632.
—— of pellagra, 461, 462, 463.
—— of martyrology, 53.
—— of stupidity, with suicidal impulse, 615.
—— of homicidal insanity, 331—333, and 604—608.
—— of impulsive insanity, 612.
—— of insanity, with analysis of the blood, 438, 439, 445—454.
—— incurable, source of, 73.
—— of pellagra, 461.
—— of feigned insanity, by Dr. Conolly, 279.
—— of eleven attacks of puerperal insanity in same person, 151.
—— interesting, from report of Suffolk lunatic asylum, 399.
—— of insanity, proportion of arising from puerperal causes, 140.
—— of puerperal insanity, 291, 294.
—— of idiotism, recovery from, in consequence of wound in head, 158.
—— of religious insanity analyzed, 232, 238, 243, 244.
Cataleptic state, the, 525.
Case of will, contested on plea of insanity, 486.
Cattell, Dr., on insanity, 430.
Causes of death classified, 116.
—— moral, in puerperal mania, 146.
—— predisposing of insanity, Feuchtersleben on, 259.
—— occasional, of, 260.
—— physical, of, 260.
—— apparent and assigned, of cases of lunacy, discharged cured from Bethlem hospital, 403.
—— exciting, of general palsy of the insane, 348.
—— predisposing, 346.
Cautions in the chamber of the dying, 122, 123.
Chamber of the dying, cautions regarding, 122, 123.
Charenton, royal asylum, description of, 110, 111.
Charenton, royal asylum, board paid at, 111.
—— restraint not altogether discontinued there, 111.
Check, salutary, proposed, affecting the character of attendants on lunatics, 34.
Child, case of mania in a, 317.
Children, destitute, in metropolis, 491.
—— precocious, fatality of, 499.
—— predisposed to insanity, alterations in physical forms of cranium, 496.
—— treatment required for, 496, 497, 498.
Christ, death of, on the physical cause of, by Dr. Stroud, 48.
—— physically considered, 54, 55.
—— immediate physical cause, rupture of the heart, illustrated, 55, 56.
Christianity, its doctrines, 557.
Cicero, on intuitive sense of right and wrong, 211.
Clarke, Dr. Adam, his sensations from submersion, 118.
Classes of society, exposed to insanity, Dr. Browne on, 392, 393.
—— educated, asylum for, Dr. Conolly on, 74.
Cline, case by, illustrating effects of physical injury to the head, 157.
Clinical facts and reflections, by Thomas Mayo, M.D., 38.
Clothing, the body clothes of lunatics, 35.
—— the bed clothes, 35.
Coercion in insanity an exception to the rule, not the rule, 35.
Commission in Lunacy on Miss A. Collings, 472.
—— in Paris, 477.
—— on two sisters, 318.
Commissions in Lunacy, 184.
Commissioners of insanity, inefficiency of, 26.
—— powers of, enlarged and extended by Bill of 1828, 26, 27.
—— metropolitan in 1844, 27.
—— duties of, 27, 28.
—— members of present commission, 28.
—— difficulties of their duties, and extreme delicacy of, 38.
Comparative anatomy of brain, and development of, 224.
Concealment of delusion, case illustrating, 382.
Conolly, Dr., case of absence of one of the hemispheres of the brain, 223.
Conscience, natural, artificial, 490.

Construction, the, and government of lunatic asylums, by Dr. Conolly, 71; commendatory notice of, 71; importance of the subject, 72; general construction of, 75; objections to large dormitories, 76.
Contagion of mental diseases, 408.
Control, self, necessity of, 495.
Convicts, six appalling histories of, 205, 206.
Convulsionnaires, account of, 419, 420.
Cooper, Sir Astley, on singular case of restoration of consciousness, 157.
Cornier, Henriette, case of, 612—614.
Correspondence, letter to the editor, 328.
——— from Paris, 466—469.
Correspondents, 192, 487, 488.
Counter-irritation in puerperal mania, 288.
Cour d'Appel at Paris, 634.
Cowan, Dr., on spiritualism, 12.
Cowper, attempts suicide by suspension, 119.
——— anecdote of, 237.
Crichton institution, report of, 389.
Criminal cases, monomania, plea of in, 483.
Criminal lunatics, proposed legislative enactment regarding, 182.
Crowe, Cath., on night side of nature, 512.
——— vagaries of her work, 512, 515, 516, 518, 522, 525, 526, 529.
Crucifixion, remarks on by Origen, 53, 54.
——— lingering nature of death from, 52.
Cruveilher, important remark of, 224.
Cullen, anecdote of in his last moments, 118.
Cumberland asylum (Dunston Lodge), report of, 395.
Curability of insanity, influenced by seasons, 62.
Cures, average proportion of in lunatic asylums, 73.

Damerow, Dr., on influence of music on the insane, 167.
Dance, St. John's, description of, 411, 412.
Dancing mania of eleventh and succeeding centuries, 411.
——— plague at Strasburg, 413.
——— presumed cause of, 414.
David, King, the feigned insanity of, 278.
Death, apathetic, how it arises, 120.
——— from crucifixion, lingering nature of, 52.
Death classification of causes of, 116.
——— considerations on, 113, 114.
——— not always the result of material causes, 116.
——— galvanization, infallible test of, 566, 567.
——— physiology of, by Mr. Dendy, 112.
——— proportions of from insanity, difference between males and females, 65.
——— signs of, 565.
——— uncertain character of, 116, 117.
Delusion, on method of cure of, 507.
Demande en Interdiction, 477.
Demonomania, 470.
Demon of revolution, mania, 423.
Dendy on the philosophy of mystery, 512.
Depression of skull, case of inducing monomania, cured by the trephine, 156.
Derangement, mental, Dr. Seymour on, 1.
——— form of, peculiar to sexagenarians, described by Dr. Seymour, 9—11.
Development of insanity, causes which favour in prison, 166.
Diagnosis of feigned insanity, from mania, 281.
——— dementia, 282.
——— monomania, 283.
——— melancholia, 283.
Dietary of lunatics, 35.
——— highly liberal scale of, 36.
——— of Bicêtre, before and after the Revolution (1792-93), 78, 79.
——— of Middlesex Lunatic Asylum, 375.
——— in puerperal insanity, 291.
Digitalis, action on the brain, 306.
Diseases, mental, statistics and pathology of, by Dr. Webster, 57.
Disease of brain and of ear connected, 189.
Disease, simulation of, amongst the insane, 393, 394.
Disinclination to food, consequent on disease of the stomach, 11.
Dissections of cases of puerperal insanity, general result of, 144.
Dissolution, pangs of, modified by state of mind, 120.
——— exemplified by the deaths of the martyrs, 120.
Doctrine, judicial, as to sense of right or wrong, fallaciousness of, as a test, 205.

Doctrines of psychology, present form of, 252.
Dormitories, large, objections to, by Dr. Conolly, 76.
Dreams, prognosis from, and indications of, 255, 256.
——— their mental and moral aspects, by John Sheppard, 512.
Dreaming, the physiology of, 512.
——— Evelyn's pathology of, 519, 520.
Drugs, certain effects of, and symptoms of diseases of the brain, analogy between, 294—298.
Duration of the insane state, how it affects the result, 66.
Duties of attendants on the insane, by Dr. Conolly, 84—86.
Dundee Lunatic Asylum, report of, 377, 580.
Dunston Lodge Asylum, report of, 395, 396.
Dying, last physical phenomena of, 123.
—— the chamber of, cautions in, 121—123.
—— the general treatment of, 123.
—— treatment of, by Mr. Dendy, 112.

Ear, disease of, connected with cerebral disease, 189.
Ecstatic state, from use of cannabis sativa, 298.
Edinburgh Lunatic Asylum, report of, for 1847, 378—383.
——— causes of diseases in cases admitted, 380.
——— pathological appearances of, summary of, 382.
Education, controlling power of, Dr. Feuchtersleben on, 259.
——— imperfect, 490, 491.
——— imperfections in, induce insanity, 493, 494.
Effects, bad, from over exercise of one faculty of the mind, 246.
Electricity, relation of to vitality, 114, 115.
Embryo, animation of theories on, 563, 564.
Emetics in puerperal mania, 286.
Emille (Charles), an idiot, state and history of, 104.
Employment of lunatics at Hanwell, 82.
England, condition of insane in, 22.
Enthusiasm, religious, and dogmatism, errors of, 240, 241.
Epidemic insanity, 406.
Epidemics, religious, mode in which they originate, 242, 243.
Errors of the age on religion, by Dr. Ideler, 229.
Esquirol on hereditary insanity, 270.
Ether vapour, Dr. Taylor on, 428, 429.
Ether, administration of, in treatment of insanity, 168.
Exhaustion, chief source of danger in puerperal insanity, 150.
Extracts and varieties, 334.

Fallacy attending the estimation of the proportion of cures from change in diet of the insane, 79.
Falsification of statistical tables, 57.
Falret and Voisin, private lunatic asylum of, 110.
Falret, remarks on restraint, 105.
Family, whole, swept away by suicide, 271.
Fanaticism, injurious consequences of to true religion, 230—232.
Farm for lunatics at St. Anne, 110.
Feigned insanity, notes on, 277.
Feuchtersleben on morbid appearances of insanity, 258.
Feelings, psychical effect of, as agents for cure of insanity, 504.
Food, quantity of, allowed to lunatics, 80.
—— full statement of kind and amount of at Hanwell lunatic asylum, 80, 81.
Fodere, biographical notice of, 337.
Folly, treatment of, 507.
Foville, chief physician of Charenton, 111
Foxglove, action of on the brain, 306.
France, criminal statistics of, 657, 658.
Frederick William of Prussia, moral insanity of, 45.
French, fancies of, regarding the English system of non-restraint, 105, 106.
Friends, Retreat of, at York, fifty-first report of, 404, 405.
Functions, cerebral, singular affection of, during pregnancy, 131.

Gall and Spurzheim, well merited encomium on, by Solly, 16.
Gangrene of lungs frequent among insane, 469.
Greeks, lycanthropy among the, 257.
Guy, Professor, on frequency of pulse as diagnostic of mania, 281.

Hachich, singular action of, 297—301.
Halford, Sir Henry, his opinion of value of medical treatment of insanity, 1.
Hallucinations and monomania, difference between, 585.
——— universality of, amongst almost all classes, 586.

Hallucinations, of sense of hearing most common, 589.
——— cases of, 589, 591.
——— and mental illusions, distinctions drawn between, 593, 594.
——— bandaging the eyes, effects of in, 595
——— of smell, case of, 599.
——— of touch, case of, 600.
Hanwell asylum, kind and quantity of food, tables of, 80, 81.
——— history of employment of lunatics at, 82.
——— non-restraint system at, Report of Magistrates of Middlesex on, 106, 107.
——— report of for 1848, 374.
——— patients and attendants, number of, 375.
Head not always symmetrical, 225.
Heads of the insane, or physical character of, by Dr. Brigham, 90.
Hereditary predisposition to insanity, by Dr. Brigham, 91.
Hints, practical, on the moral, mental, and physical training of girls at school, by Madame de Wahl, 489.
History of Medical psychology, 248—250.
Holland, Dr., on phrenology, 15.
Homicidal mania, without delirium, 568—570.
Homicidal insanity, 330.
——— case of, 331—334.
Hope, disappointed, features and traits of the effect on the mind, 197.
Hungerbuhler on lunatic asylums in Switzerland, 92.
Human species, natural history of, by Colonel Smith, 571.
Hunter, John, summary notices of the extent of his knowledge of the nervous system, 13.
Hunter, William, his imperfect notions of the nervous system, 14.
Hydrophobia, 344.
Hygiene, mental, importance of, 495.
Hypochondriasis, remarks on, 201, 202.
Hysterical mania, case of, described, 130.
Hysteria, Feuchtersleben on, 256.

Idea, definition of, 222.
Ideler, Dr., on religious insanity, and religious errors of the age, 229.
Ideler, Dr., Transcendental character of his work, 231.
Idiots of the Bicêtre, Dr. Sigmond on, 124.
——— course of their studies, 125.
Idiots, extraordinary calculating power of some, 127.
——— song of the, 103.
——— reunion of at Bicêtre, 103.
——— asylum proposed for, 190.
——— treatment of at Massachusets, 623.
——— at Paris, 623.
Idiotic female, morbid inspection of, 334.
Idiotism, cured by a blow to the head; several cases of, 158.
Illusions, remarks on, 521—523.
——— of disordered vision, 523.
Imitation, influence of, in epidemics, 406—407.
Impairment of mind, Dr. Browne on, 389, 390.
Imprisonment, solitary, defended by Dr. Basting, 159—166.
——— at Glasgow, 165.
Impulsive insanity, 609.
——— case of, 610, 611.
——— Dr. Baillarger on, 615.
Incubation of insanity, Dr. Winslow on, 132.
Incurable, hospitals for, warmly defended by Dr. Conolly, 74.
——— his ingenious and sound arguments in favour of, 74.
Influences, mental, Dr. Moore on, 366.
Influence, morbid, of mind on body, and *vice versa*, 253—254.
Injuries, physical, of brain, induce all varieties of mental disease, 155.
Injury, accidental to head of insane patient, restores the reason, 158.
Innate idea on, the perception of right and wrong, 211.
Innocent the Eighth, bigotry of, 93.
——— notorious infamy of his bull, 93.
Insane, morbid appearances in the brain; are they a cause or consequence of the insane state? 64.
——— diet of influence on, by Dr. Thurnam, 68.
——— chronic general palsy of, by Dr. Rodrigues, 345.
——— construction and government of hospitals for, 71.
——— status of in society, to be consulted in the position and form of the asylum, 74.
——— physical character of their heads, 91.
——— dissections of, 108; made at Bethlehem, analysis of 63.
——— deaths from, difference between male and female, 65.
——— pulse of, by Dr. Brigham, 90.
——— number of in Norway, 314.

Insane state, kind of, in Norway, tables of, 314.
——— liable to caprices during pregnancy, 132.
——— administration of medicines to, Dr. Brown's observations on, 395.
——— music, its influence on the, 167.
——— imperfect state of treatment of, in Switzerland, 93.
——— reunions amongst, at Salpêtrière, 102.
——— state, general, of, at Salpêtrière, 102, 103.
——— the, condition of, forced upon the legislature, 23.
——— aggregate amount of, in the united kingdom, 25.
——— poor, parliamentary commission appointed to investigate, 25.
——— over, summary and tyrannical power of justices formerly, 25.
——— visitors of, precarious benefits of, 25, 26.
Insanity, administration of ether in treatment of, 168.
——— development of, Pennsylvanian or cell system favourable to, 159.
——— importance of medical treatment in, Sir H. Halford on, 1.
——— curability of, as influenced by the seasons, 62.
——— is it a disease of the blood? 541, 557.
——— arguments in favour of, and against, balanced, 545—551.
——— Dr. Cattell on, 430.
——— purely physical, 430.
——— classification of, considered legally, 214.
——— feigned, notes on, by Dr. Robertson, 277.
—————— causes of, 278, 279.
—————— extreme difficulty of diagnosis from real, 279, 283.
——— from critical periods in female life, 129.
——— case of, occurring during period of catamenial discharge, 129, 130.
——— after labours, 133.
——— classes exposed to, Dr. Browne on, 389, 390.
——— hereditary, transmission from father or mother, 270—272.
—————— apt to appear at same periods in children as in parents, 274.
——— homicidal, plea of, 335.
——— intermittent, singular case of, 377.
——— from protracted lactation, 137.
Insanity, symptoms peculiar to, 137—139.
——— cases of, 138, 139.
——— liability of sexes to, 69.
——— remarks on, by editor, 69, 70.
——— medical jurisprudence in, 318.
——— moral treatment of, not opposed to physical means, 72.
——— moral, ambiguous nature of term, 201.
——— partial, features of, 196.
——— progressive, generally, 196.
——— waywardness and eccentricity of early stages of, 196.
——— not produced by true religion, 245.
——— new view of, the duality of the mind, by Dr. Wigan, 218.
——— outlines of, lectures on, by Sir Alexander Morison, 579.
——— religious, illustrated by cases, 229.
——— peculiar interest attached to, 230, 231.
——— genuine instinct of self-preservation lost in, 209.
——— puerperal, on the causes, symptoms, and treatment of, by Dr. James Reid, 128.
—————— symptoms of, described, 134—136.
—————— periods of attack, 136.
—————— inferences of Esquirol, 136.
—————— hereditary predisposition in, 136.
—————— causes of, 143.
—————— connected with irritability, 144.
—————— predisposing and immediate, 147.
—————— prognosis of, 147.
—————— duration of the malady, 147.
—————— different authorities quoted, 147, 148.
—————— fatal termination of, 149.
—————— Dr. Macdonald on, 531.
—————— causes of, 535, 536.
—————— peculiarities of, 537.
—————— prognosis of, 537.
—————— venesection in, 538.
—————— sleep, how procured in, 539.
—————— when wine proper, 540.
—————— chronic, how to be treated, 540.
—————— case of, treated by acetate of morphia, 7.
—————— from fright in pregnant state, treatment of by acetate of morphia, 8, 9.

Insanity, puerperal, prevention of, Dr. Macdonald on, 532.
——— proportion of, compared with other forms of insanity, 533.
——— period at which it occurs, 534, 535.
——— germ of, frequently found in early education, 492.
——— tested by science, Dr. Burnett on, 541.
——— political and epidemic, 406.
——— proximate cause of, observations on, by Mr. Shepherd, 541.
——— plea of ground for contesting a will, 486.
——— statistics of, 342.
——— observations and essay on the statistics of, by Dr. Thurnam, 57.
——— hereditary predisposition to, Dr. Brigham on, 91.
——— general view of asylums for, on the continent, 94, 95.
——— as influenced by the seasons, tables of, from Bethlem Hospital, 62.
——— curability of, as affected by the seasons, 62.
——— general course of, how modified by the weather, 63.
——— constitutional case of, detailed, 199.
——— result of, how modified by the previous duration of the attack 66.
Instinctive insanity acknowledged by the French authorities, *Marc, Esquirol, Pinel*, 47.
Instruction of medical pupils in mental diseases, 185.
Intellect, the human, 340.
Intellectual powers improved by the trephine, case of, 158.
——— instinct of a maniac, 341
——— observations on, extracted from Mr. Barlow, 47.
Ireland, state of lunatic asylums in, 151.
——— discreditable condition of, 151.
——— bad effects of irregular medical visits in, 153.
Irritation counter, in puerperal mania, 288.
Irritability of cerebro-spinal system cause of puerperal insanity, 144.
Italy, Tarantism in, 416—418.

Jacobi, jubilee of his doctorate, 338.
Jebb, Bishop, on parallelism of Scripture, 227.
Jones, Bence, on diathesis of phosphatic salts in nervous diseases, 188.
Judicial, the, criterion of insanity inadequate in cases of partial insanity, 193, 194.
Judicial test of moral perversion, can it be fairly applied? 207.
——— inapplicable in its ethical sense, 212.
——— psychology, 511.
Juries, present constitution of, totally incompetent to offer a correct verdict on plea of insanity, 39.
Jurisprudence, medical, in relation to insanity, 169, 634.
Jury, grand, charge of Baron Rolfe to, analytical review of 193.
Jubilee of Jacobi's doctorate, 338.

Keeper, term of, repugnant and objectionable, 82.
Kent lunatic asylum, report of, and statistical tables, 397—399.
Knowledge, slow progress of, 229.

Labour, insanity after, 132.
Lactation, insanity from, 137.
Lady and Beggar, singular anecdote of, 203, 204.
Lancaster lunatic asylum, report of, 376, 377, 580—582.
Lawrence, Mr., dissections of cases in Bethlem Hospital, 63.
Lectures on psychology, analysis of, 247.
Letter from C. Pearson, Esq. to the editor, 182.
Leubuscher, on hereditary transmission of insanity, 264.
Liability, different of, the sexes to insanity, 69.
Life assurance; suicide, 328.
Living generously strongly advised in insanity by Dr. Conolly, 78.
——— effects of poor diet contrasted with, 78.
Luke's, St., description of, in 1815, 1816, 29, 30.
Lunacy, 471.
——— condition of, in England, 22.
——— commission in, on two sisters, 318.
——— commissioners in, 184.
Lunatics, criminal, proposed legislative enactment in reference to, 182.
——— tables of, confined in Bethlem, and the causes, 403.
——— helpless and hapless state of, 22.
——— civil condition of, 89.
——— how disposed of in England and Wales, 23.
——— estimate, tabular, of, and statement of expenses, 24.
——— pauper, their condition overlooked, 73.

Lunatics, average expense of patients in public and private asylums, 73.
——— body clothing of, 35.
——— bed clothing of, 35.
——— dietary of, 35.
——— parishes often at fault, neglecting the interests of, 36.
——— at Hanwell, meals of, how conducted, 81.
Lunatic asylum, well regulated account of, 32, 33.
——— of moderate size recommended, 67.
——— supposed influence on mortality, 67.
——— and hospitals, construction and government of, by Dr. Conolly, 71.
——— enormities of, prior to 1815, from report, 28.
——— of York, history of, 68.
——— State, near Utica, fourth annual report of, 87.
——— private, of Falret and Voisin, 110.
——— Parisian, notes on, 100.
——— in Switzerland, 92.
——— state of, in Ireland, 151.
Lycanthropy of the Greeks, 257.

Magic, philosophy of, 512.
Malformation of brain, case of, by the late Dr. Wigan, 306—309.
Malleus Maleficarum, work referred to, 93.
Mansour, his tale, 301.
Mania, more fatal to men than women, 60.
——— homicidal, case of, 605—608.
——— hysterical, 130.
——— intellectual, instinct of a, 341.
——— religious, portrayed, 198, 199.
——— treatment of, 508—510.
Martyrology, cases of, 53.
Material causes not always essential to death, 116.
Mayo, Dr., clinical facts and reflexions by, 38.
——— peculiar remarks as to modified punishments in cases of imperfect development of the moral principle, 48.
Meals of lunatics at Hanwell Asylum, how conducted, 81.
Medical intelligence, 188.
——— jurisprudence in relation to insanity, 169, 318, 472, 634.
——— psychology, principles of, by Dr. Feuchtersleben, 247.
——— difficulties attending the study of, 248.
Medical society of London, 189.
——— treatment of insanity, by Dr. Seymour, 2.
Melancholy of Augustus, cured by lettuce, 5.
——— moral principle in, greatly weakened or impaired, 48.
Memory, 344.
——— case of remarkable failure of, 471.
Menorrhagia, insanity occurring during, 129, 130.
Mental affections, principles of prognosis in, by Dr. Feuchtersleben, 262, 263.
——— alienation, great number predisposed to, 409.
——— alienation; arson, 476.
——— cultivation, Dr. Moore on, 373.
——— derangement, Dr. Seymour on, high character of his work, 1, 2.
——— qualities, transmission of, 575.
——— diseases, lower modes of, peculiarly hereditary, 272.
——— instruction of medical pupils in, 185.
——— faculties, and paralytic affections, 343.
——— influences, 366.
——— illustrations of, 370, 371.
——— life, influence of sexual functions on, 254, 255.
Michea, Dr., on the blood in the neuroses, 436.
Middlesex lunatic asylum, dietary of, 375.
——— magistrates, report upon non-restraint system at Hanwell, 106, 107.
Mind, the mysterious nature of, 218, 219.
——— a first principle, or entity indivisible, 220.
——— one faculty of, unduly exercised, dangerous consequence of, 246.
——— culture of, as a means of cure of insanity, 501—503.
——— duality of, Dr. Wigan on, 218.
——— disease of, always connected with bodily disorder, 215.
——— human, observations on, 209—211.
——— use of the body in relation to, 489.
——— effect of the weather on, 342.
——— world of, full of wonders, 371.
——— morbid influence of, on the body, 253, 254.
——— pathology of, 256—258.
Mirror, the Morningside, extract from, 582.

Miscellaneous, 340, 469, 579.
Modifications of insanity arising from cerebral injury, 155.
Monomania, connected with pilfering, 205.
——— plea of, in criminal cases, 483.
——— case of, with remarks, 641, 652.
——— of grandeur always connected with palsy, 460.
——— with fear, 470.
——— does not necessarily involve moral irresponsibility, 45, 46.
Moore on the power of the soul over the body, 366.
——— high character of his work, 368.
Moral insanity, ambiguity of the term, 201.
—— mania, 342.
—— perversion, judicial test of, can it in any case be applied? 207.
—— principle does exist, 42.
——— how far affected in the different varieties of insanity, 43.
——— exemplified in life of Frederick William of Prussia, 45.
—— responsibility, Dr. John Davy on, 40.
——— Mr. Samson on, 40.
——— British Review on, 40.
——— analysis of opinions advocated in British Review, 41, 42.
Moreau, Dr., of Tours, on action of cannabis indica, 297, 301.
Morison, Mr., case of mania in a child six years old, 307.
Morphia, acetate of, now recommended by Dr. Seymour, 5.
——— muriate of, in suicidal cases, by Dr. Seymour, 4, 5.
——— modus operandi of, 6.
——— objections against, founded on law of habit, stated and answered, 6.
——— cases illustrative of, action of, 6, 7.
Mortality from mental diseases, connexion with the seasons, 62.
Music, influence on the insane, 167.
Murder, charge of, acquittal on ground of insanity, 480.
——— remarks on, 481—483.
——— charge of, acquittal on ground of puerperal insanity, 478.

Nares, Archdeacon, on phrenology, 15.
Nature, Night side of, 512.
Nebuchadnezzar, case of perfect insanity, 195.
Nervous system, imperfect ideas of the Hunters on, 13, 14.
Nervous system, summary view of progress of discovery in, 14.
——— and mind, development of, proportionate, 16.
——— development of, through whole system of animated nature, 16.
——— diseases, excess of phosphatic and earthy salts in, 188.
Neuroses, on the blood in, by Dr. Michea, 436.
New view of insanity, by Dr. Wigan, 218.
Notes on feigned diseases, by Dr. Robertson, 277.
——— on the Parisian lunatic asylums, by Dr. Stubb, 100.
Norway, number of insane in, 314.
Nosology, or arrangement of mental diseases, 261, 262.

Observations and essays on the statistics of insanity, by Dr. Thurnam, 57.
Offspring, how influenced by character of parents, 267.
Opium, reason why inefficient in mania, 5.
——— habitual use of by lower orders, probably cause of increase of insanity, 145.
——— much used by the public as a stimulant, 145.
——— description of its effects, 396, 397.
Origen, remarks of, on the crucifixion, 53, 54.
Ovenston, John, trial of, 169.
——— address of Mr. Clarkson in favour of, 173.
——— remarks on, by the Editor, 176, 177.
——— opinion of Dr. Conolly, 176.
Palsy, general chronic, of the insane, by Dr. Rodrigues, 345.
——— general predisposing causes to, 346.
——— exciting causes of, 348.
——— early symptoms of, 348.
——— progress of symptoms of, 349, 352.
——— premonitory symptoms of, sometimes intermittent, 349.
——— character of insanity associated with, 351.
——— duration of, 352.
——— prognosis of, 352, 353.
——— recovery from, very rare, 353.
——— complications of, 353.
——— pathological appearance, 354—361.
——— uncertain state of pathological views, 359—362.

Palsy, treatment of, 362—365.
——— general, accompanied by the monomania of grandeur, 460.
Paralysis of the tongue induced by passion, 334.
Paralytic affections in connexion with mental faculties, 343.
Parallelism of Scripture, 227.
Paris, correspondence from, 652, and 466—469.
—— cour de appel at, 634.
—— treatment of idiots at, 623.
Parisian lunatic asylums, notes on, 100.
Parents, how they affect their offspring, 267.
Passions, sexual, influence in religious epidemics, 242, 243.
Pathology of insanity, by Dr. Seymour, 3—5.
——— of the mind, 256—258.
——— and statistics of mental diseases, by Dr. Webster, 57.
Patients, lunatic, average cost of, in private and public lunatic asylums, 73.
Pauper lunatics, their condition forgotten, 73.
Pearson, C., Esq., M.P., letter to the editor, 182.
Pellagra, on the, by Dr. Baillarger, 460.
——— peculiarities of, 464, 465.
Periodoscope of Dr. Smith, 657.
Penitentiary, system of, influence of inducing insanity, 458, 459.
Pennsylvanian, or cell system, is it favourable to development of insanity? 159.
Periods, critical, in females occasional causes of insanity, 129.
Peter the Hermit, the magic of his voice, 410.
Philosophy of mystery, 531.
Phrenitis, diagnostic marks of, from puerperal insanity, 137.
——— symptoms of, 136, 137.
Phrenology, Archdeacon Nares on, 15.
——— Dr. Holland on, 15.
Phosphatic earthy and alkaline salts, in relation to nervous diseases, 188.
Physical cause of the death of Christ, Dr. Stroud on, 48.
Physiology of death, 112.
——— of psychiatrics, 253.
Physical nature of insanity, Dr. Cattell on, 430.
Pilfering, monomania connected with, 205, 206.
Plea of insanity, 194, 335.
——— grounds of according to the fifteen judges, 39.
Plea of insanity, proposal of trying the guilt by one jury—the insanity by another, 40.
Poisons, in relation to medical jurisprudence and medicine, 425.
Poison, sausage, Dr. Taylor on, 426, 427.
Poor, insane, comparatively small number of, provided for, 73.
Pregnancy, effects of frights during, 133.
——— singular affection of cerebral functions during, 131.
Principle of spiritualism, Dr. Cowan on, 12.
Priest and the physician, 557.
Prisons, causes in, which may favour the development of insanity enumerated, 166.
Prize question of the university of Berlin, 169.
Prognosis and indications from dreams, 255, 256.
Propositions of Dr. Wigan on the duality of the mind reduced to four heads, 222.
Prussia, Frederick William of, a case of moral insanity, 45.
Psychological medicine, report of, by Dr. Robertson, 579.
Psychology, medical, 501.
——— Dr. Feuchtersleben on, 247.
——— present doctrine of, exposition of, 252.
Predisposition, hereditary, to insanity, Dr. Brigham on, 91.
Public institutions, superiority over private, 510, 511.
Puerperal insanity, on the causes, symptoms, and treatment of, by Dr. James Reid, 128.
——— hereditary predisposition to, 136.
——— proportion of, to total cases of insanity, 140, 143.
——— causes of, 143.
——— duration, 144.
——— convulsions and puerperal mania induced from same cause, 144.
——— influence of moral causes in producing, 146.
——— prognosis of, 147.
——— duration of the malady, 147.
——— Denman, Dr., his observations on, 147, 188.
——— fatal termination of, 149.
——— general mortality from, comparatively low, 150.
——— liability to after attacks, 151.
——— treated by acetate of morphia, case of, 7.
——— anodynes in, 286—288.
——— moral treatment of, 286, 289.

Puerperal insanity, cases of, 291, 293, 294.
——— extreme disposition to suicide in, 290.
——— dietary in, 291.
——— bleeding in, 285.
——— purgatives in, 286.
Pulse of the insane, by Dr. Brigham, 90.
—— frequency of, is diagnostic of real mania, 281.
—— table of, by Drs. Leuret, Mitivié, and Professor Guy, 281.
Punishment, modified degree of, under certain cases of imperfect development of moral principle, Dr. Mayo on, 48.

Qualifications of attendants on lunatics, Dr. Conolly on, 84.
Questions, prize, of, at the university of Berlin, 169.
Quietude, importance of, in treatment of puerperal mania, 289.

Rambouillet, Marquis de, and Marquis de Percy, anecdote of, 597, 598.
Reason, faculty of, not gone in mania, 200.
——— and appetites, mutually opposed, 492.
Reflective powers, how influenced by hallucinations, 602—604.
Reid, Dr. James, on the causes, symptoms, and treatment of puerperal insanity, 128, *et seq.*
Religious insanity, cases of detailed, 232, 238, 243, 244.
——— prophylaxis of, 236.
——— less frequent among Romanists than Protestants, 226.
——— baneful influence of fanaticism on, 230, 231.
Religious mania, sketch of, 198, 199.
——— insanity, prevention of, 246.
Religion, true, does not induce insanity, 245.
Remarks on hereditary transmission of insanity, 264.
Removal to lunatic asylum generally too late, 37.
Report of Commissioners in lunacy to the Lord Chancellor, 22.
Report, Fourth Annual, of State Asylum, New York, 87.
Reports of Lunatic Asylums, 374—405.
Aberdeen, 387.
Bedford, 396.
Bethlem, 401.
Crichton, 389.
Cumberland, 395.
Dundee, 377.
Reports of Lunatic Asylums—*continued.*
Edinburgh, 378.
Hanwell, 375.
Friends' Retreat, 404.
Kent, 397.
Lancaster, 376.
Staffordshire, 384—387.
Suffolk, 383, 399.
Resemblance, physical, proof of transmission, 267, 268.
Respect, Dr. Conolly on, due to the insane, 81, 82.
Restraint, average weekly number of patients under, in Bethlem for seven years, 109.
——— Falret on, 105.
——— non system of, Report of Middlesex magistrates at Hanwell, 106, 107.
——— remarks on, 108.
——— still employed at Bicêtre and Saltpêtrière, 107.
——— often exciting cause of suicide, 109.
——— system of, not attended with additional security, 61.
——— non, fancies of French regarding the English system of, 105, 106.
——— Dr. Conolly on, 76.
——— cases illustrative of, 77.
——— personal, of lunatics, diminished, good results from, 34.
Retreat at York, statistical tables connected with, medical and economical, 404, 405.
Reunions amongst insane at Salpêtrière, 102.
Right and Wrong, is there a principle of, argued? 39, 40, *et seq.*
——— consciousness of, Dr. L. Robertson on, 38.
——— often arbitrary terms, 212, 213, 214.
——— no proof of sanity, 214.
Robertson, Dr., on the application of the trephine to the cure of insanity, the result of injury to the head, 155.
——— on feigned insanity, 277.
Rodrigues, Dr., on chronic general palsy of the insane, analytical review of, 345.
——— summary opinion of the work, 366.
Rolfe, Baron, charge to the jury in case of boy Allnutt, analytical review of, 193.
Rural walks recommended, 77.

Salpêtrière, reunions amongst the insane at, 102.

Salpêtrière, duration of cases of puerperal mania there, 149.
——— general disposition and arrangements of, 100—102.
Salverte on the philosophy of magic, 512.
Scene, almost incredible, at Castillane, in Lower Alps, 653.
School of Idiots, day's duties detailed in, 625, 626.
Scripture, parallelism of, 227.
Season of the year, relations to the insane state, as shown by the tables of Bethlem Hospital, 62.
——— when mortality greatest, 62.
Selection of site for lunatic asylums, 66, 67.
Seguin on the treatment of idiots, 624.
Sepulture, premature cases of, 117.
Sexes, relative liability of, to insanity, 69.
Sexual function, influence of, on mental life, 254, 255.
Seymour on mental derangement, 1.
——— refers to two morbid actions a plus, a minus condition of the brain, 2, 3.
——— believer in functional disorder independent of actual cerebral disease, 3; question argued, 3, 4.
——— on use of acetate of morphia in suicidal cases, 4, 5.
——— on pathology of insanity, 3, 4.
——— on peculiar form of derangement of the mind in sexagenarians, 9—11.
Sheppard on dreams, in their mental and moral aspects, 512.
——— on the proximate cause of insanity, 541.
Sigmond, Dr., on the idiots of the Bicêtre, 124.
Simulation of disease among the insane, 393.
Skae, Dr., on ether in insanity, 383.
Sketch of principal asylums of Europe, 252.
Skull, depression in case of, resulting in monomania, cured by the trephine, 156.
Sleep and death, considerations on, 112, 113.
Societies, debating, in lunatic asylums of New York, 91.
Society, medical, of London, 189.
Solitary system, advocated by Dr. Basting, 159.
Solly on the brain, 13.
Solly on the brain, analysis of, 17—19.
——— on the pathology of the brain, 20.
——— makes four divisions of the pathology, 20.
——— reflections on his pathological views, 21, 22.
Somnambulism depending on the use of belladonna, 304.
Song of the idiots, 103.
Souard, St., history of an endemic monomania in a battalion of French soldiers, 421.
Soul, power of, over the body, Dr. Moore on, 366.
Species, human, natural history of, by Colonel Smith, 571; analysis of book, 571, 575; contends for more than one origin of the species, 572.
Spiritualism, Dr. Cowan on, 12.
Staffordshire Lunatic Asylum, reports and tabular statements of, 383—387.
Statement, tabular, of duration of puerperal insanity at Bethlem hospital, 149.
——— of Irish lunatic asylums, 154.
Statistical tables, falsification of, 57.
——— importance of being drawn up on an uniform system, 66.
Statistics of insanity, and observations on, by Dr. Thurnam, 57.
——— and pathology of mental diseases, by Dr. Webster, 57.
——— criminal, of France, 657, 658.
Stewart, H. Keith, Esq., commission in lunacy on, 184.
Stephens, Sir George, on plea of monomania in criminal cases, 483.
Stomach, actual disease of, cause of certain hallucinations, 11.
Strasburgh, dancing plague at, 413.
Stramonium datura, effects of, on the function of the brain, 301, 302.
Stroud, Dr., on the physical cause of the death of Christ, 48.
——— reflections suggested by the work, 49, 50, 51.
Stubb, Dr., notes on the Parisian lunatic asylums, 100.
Stupidity, case of, with suicidal impulse, 615.
——— of insane, obscure nature of, 618—620.
——— Baillarger on, 620.
——— Moreau on, 621, 622.
Submersion, sensations produced by, described by Dr. Clarke, 118.
——— reviving from, agony of, 119.

Suffering, writhing, and groaning, no test of, 119.
Suffolk, lunatic asylum of, report and tabular statements of, 399—401.
——— interesting case from, 399, 400.
Suicide, extremely sudden tendency to, in puerperal mania, 290.
——— Dr. Browne on, 391, 392.
——— whole family victims of, 271.
——— less frequently than formerly in insanity, 60, 61.
——— more prevalent in females than males, 61,
——— restraint, occasional exciting cause of, 109.
——— in Grand Duchy of Baden, statistics of, 315, 316.
——— table of, motives for, 316.
Suspension (hanging) not painful, 119.
Symptoms of puerperal insanity described, 134—136.
Switzerland, lunatic asylums in, 92.
Swiss Cantons, imperfect organization of lunatic asylums in, 96, 97.
——— tables from, 98, 99.
——— difference in the character of the asylums, affected by political character of the, 99.
Sympathy, agency of, as epidemics, 406.

Talleyrand, anecdote of, 586.
Tailor, singular action of belladonna on, 304.
Tasso, hallucinations of, 591, 592.
Tarantism in Italy, 416—418.
Taylor on poisons, in relation to medical jurisprudence of medicine, 425.
——— on effects of ether vapour, 428, 429.
Temperament, erotic, of Dr. Debreyne, 562.
——— sanguine, described, 560.
Termination, fatal, of puerperal insanity, 149.
Theatrical exhibitions in State Asylum of New York, 91.
Theology, moral, Dr. Debreyne on, 557.
Thurnam, Dr., observations and essays on the statutes of insanity, 57.
——— critical remarks on his works, 69.
Tobacco, action of on the brain, 306.
Tongue, palsy of, induced by passion, 334.
Transmission, hereditary, of insanity, 264.
——— of physical resemblances, testimonies of, 267.
Trial of John Ovenston for shooting, with intent, &c., 169.
Treatment, heroic, of palsy of the insane, 362.
Treatment, moral, of puerperal mania, 288, 289.
——— of the dying, 112, 123.
Trepanning, increase of intellectual power in consequence of, 158.
Trephine, use of, in case of insanity from injury to the head, 155.

Varieties and extracts, 334.
Verdicts of juries in cases of plea of insanity, altogether incompetent, Dr. Mayo's remarks on, 39.
——— difficulties attendant on, 39, 40.
Vienna, ball in the asylum at, 169.
Visitors of insane almost valueless, 25, 26.
Visitations of lunatic asylums, good effects of, 33.
——— extract from reports of commissioners of, 33.
Vitality, connexion of with electricity, 114, 115.

Walter, Dr., on compression of the carotids, 626.
Weather, effects of on the mind, 342.
Webster, Dr., on statistics and pathology of mental diseases, 57.
——— on instruction of medical pupils in mental diseases, 185.
Wigan, Dr., the late, case of malformation of the brain, and inferences, 306—309.
——— on the duality of the mind, his propositions reduced to four heads, 222.
——— supports the doctrine by cases, 223.
——— scattered and curious ideas contained in, 226.
Will contested on plea of insanity of testator, 486.
Winslow, Dr. Forbes, remarks on case of John Ovenston, 176.
——— on incubation of insanity, 132.
Women, insanity more common among than men, 60.
——— recover more commonly than men, 64, 69.
——— exceptions to the law, 65.
Wonders, the mind, a world of, 512.
World unseen, communications with, 512.

York asylum, former horrors of, 29.
York, new state lunatic asylum of, fourth annual report of, 87.
——— statistics of, since its first opening in June, 1823, 87.
——— capricious character of mortality in, 88.

